# Editorial: Computational and Experimental Approaches in Multi-target Pharmacology

**DOI:** 10.3389/fphar.2017.00443

**Published:** 2017-06-30

**Authors:** Thomas J. Anastasio

**Affiliations:** Computational Neurobiology Laboratory, Department of Molecular and Integrative Physiology, Beckman Institute for Advanced Science and Technology, University of Illinois at Urbana-ChampaignUrbana, IL, United States

**Keywords:** polypharmacy, drug combination, drug repurposing, synergy, systems biology, multifactorial process, computer modeling, high throughput screening

## Multi-target treatments for multifactorial diseases

Picture yourself in the cockpit of the new Boeing™ 737 MAX airliner, or at the control console of a new American Atomics™ nuclear reactor. You are in charge, and hundreds to thousands of lives depend on your skillful control of a very complex man-made system. Fortunately, these systems are highly automated, so you need do little more than watch a few displays. Then the airliner goes into a nosedive or the reactor overheats, and the computer fails! You need to take manual control to avoid disaster. To make matters more interesting, imagine that, in order to control that nosediving airliner or that overheating reactor, you have access *not* to all the controls, or even to several controls, but to *only one* control. Can you further image that you would succeed in averting disaster?

Biomedical researchers of many stripes are engaged in battles against multifactorial disease processes that are fought within the dense jungles of very complex physiological systems. Most of them still seem to imagine that they will win the battle by using a single drug to alter the biological properties of a single drug target. How is that working out for them? Take Alzheimer Disease as an example. For decades the Alzheimer field has focused on a single peptide, the amyloid-β peptide, and has devoted vast resources to lowering it using drugs targeting its synthetic enzymes (Armstrong, 2014; Hardy et al., 2014). After all this effort we still lack effective means to halt the neurodegenerative processes associated with Alzheimer Disease. We can't even slow them down.

Increasingly, forward-thinking researchers are calling for the development of multi-target/multidrug treatments for Alzheimer Disease (Bajda et al., 2011; Leon and Marco-Contelles, 2011; Carmo Carreiras et al., 2013). I had my epiphany while creating a computational model of the metabolism of amyloid-β. When I read the literature on the effects of estrogen on this process, in order to connect estrogen with the other elements of my model, I found that this hormone targets not one but at least 10 different elements of the system that regulates amyloid-β (Anastasio, 2013). Hormones, naturally occurring interventional agents that have evolved over eons, achieve control of complex physiological systems by manipulating many system elements simultaneously. We should strive to do the same in identifying treatments for Alzheimer Disease and other multifactorial disorders.

Diseases having multifactorial etiologies include Alzheimer and other neurodegenerative diseases, cancer and cardiovascular disease, diabetes and obesity, and depression and schizophrenia. Multi-target treatments for some multifactorial diseases already exist, and multidrug regimens for AIDS, infection by drug-resistant bacteria, cancer, diabetes, and even some mood disorders are by now standard. And the hunt is on for new multi-target approaches. It is widely acknowledged that the main impediment to the design of multidrug/multi-target treatments is the failure to understand the multifactorial processes themselves. New computational models are needed that can represent the interactions among the many factors involved, and new experimental methods are needed to evaluate the validity of the models. Several recent surveys describe the current landscape (Keith et al., 2005; Boran and Iyengar, 2010; Xie et al., 2012; Reddy and Zhang, 2013; Billur Engin et al., 2014; Bulusu et al., 2016). In this Research Topic, leading experts in the area of multi-target pharmacology present their most recent new findings, new models, and new ideas, and show the way forward in the identification of new multi-target/multidrug treatments for multifactorial diseases.

## From medicinal plants to multidrug strategies

Medicinal plants are the original multidrug medicines, and many traditional treatments involve plants that have verifiable medicinal properties. For example, *Borreria verticillata* has been used traditionally in Brazil to treat pain. Silva et al. demonstrate that crude extracts of this plant do indeed have antinociceptive properties, and proceed to analyze its constituents experimentally and computationally.

Medicinal plants were discovered by trail-and-error but multi-target/multidrug therapies could be designed *de novo*. An example of a designer drug pair is the “binary weapon” of Grixti et al. in which the tumor cell toxicity of one compound is increased through downregulation of its efflux transporter by another compound. The Kell lab provides evidence that various small molecule drugs can increase the toxicity to pancreatic cancer cells of the nucleoside analog gemcitabine. In a study that unifies the traditional and the modern, Gao et al. show how protocatechuic aldehyde, a compound isolated from the Lamiaceae root used in traditional Chinese medicine, can ameliorate some of the serious adverse side effects of the chemotherapeutic agent cisplatin.

## Drug combination identification using computational brain models

Neurological and psychiatric disorders exemplify the challenge of understanding a pathophysiological process well enough to identify an effective polypharmacological treatment for it. Increasingly, computational models are being used to aid the design of effective drug combinations for the treatment of brain diseases. Geerts et al. have developed a computational model of cerebral cortex, featuring a network of many biologically realistic pyramidal neurons and interneurons. Using computational analogs of the working memory tasks that are used to assess cognitive impairment in schizophrenics, they perform *in silico* screens to predict novel drug combinations that would be effective in ameliorating schizophrenic symptomatology. In a similar vein, Neymotin et al. present a computational model of dystonia, a movement disorder associated with involuntary muscle contractions involving several interacting brain regions. They produced a computational model of these brain regions containing a multitude of biologically realistic model neurons, and use it to suggest new multidrug treatments.

## The benefits and challenges of multi-target pharmacology

Perhaps the most obvious way to strike multiple pharmacological targets is to administer multiple drugs, but major challenges in the design of multidrug treatments are mismatches in the pharmacokinetics of the different drugs in the combination. This issue is obviated using single compounds that can strike multiple targets, but finding or synthesizing such multi-target ligands pose challenges of their own. Talevi gives numerous examples of effective multi-target drugs and suggests new ways to identify more. Rastelli and Pinzi elaborate on the multi-target ligand theme and provide an overview of computational tools and related approaches for identification of promising candidate compounds.

Physiological processes are difficult to control not only because they are complex but because they adapt. Xie and Bourne lay out the challenges associated with the development of multi-target strategies to prevent tumor growth due to the resistance to anti-cancer drugs that tumors often develop.

The hoped for response to any drug combination is a synergistic interaction that enhances the desired effects of the individual drugs, or that causes new desired effects to emerge. But synergy in the biological context can occur in various ways and quantifying it is not always straightforward. Tang et al. outline the problems and suggest that the best way to describe synergy is to combine two well known methods. One possible benefit of a multidrug combination is reduction in individual drug dosage such that the desired effect arises synergistically from the combination while unwanted side effects due to individual drugs are minimized. The flip side is the potential drawback that unwanted side effects could be exacerbated, or new side effects could emerge from the combination. The ability to predict the possible side effects of novel compounds would be of value in the design of multidrug strategies, and Lopes et al. describe a new method for doing that.

From drug-resistant bacterial infections to neurodegeneration, the biomedical community faces treatment challenges that involve confronting, understanding, and ultimately manipulating disease processes of great complexity. The articles in this Research Topic direct us along many computational and experimental avenues that we can pursue in identifying multi-target/multidrug treatments for multifactorial disorders.

## Author contributions

TA served as Topic Editor for this Research Topic and also wrote this Editorial.

### Conflict of interest statement

The author declares that the research was conducted in the absence of any commercial or financial relationships that could be construed as a potential conflict of interest.
